# A giant mucinous cystadenocarcinoma of the appendix: a case report and review of the literature

**DOI:** 10.1186/s12957-016-0828-2

**Published:** 2016-03-05

**Authors:** Hiroshi Nagata, Yuji Kondo, Kazushige Kawai, Soichiro Ishihara, Shinsuke Kazama, Takako Nirei, Daisuke Soma, Jun Yamada, Eiji Sunami, Joji Kitayama, Yoshiro Kubota, Toshiaki Watanabe

**Affiliations:** Division of Surgical Oncology, Department of Surgery, Faculty of Medicine, The University of Tokyo, 7-3-1 Hongo, Bunkyo-ku, Tokyo, 113-8655 Japan; Department of Surgery, Kikkoman General Hospital, 100 Miyazaki, Noda-city, Chiba 278-0005 Japan

**Keywords:** Mucinous cystadenocarcinoma, Appendix, Mucocele

## Abstract

**Background:**

Mucinous cystadenocarcinoma is the second most common etiology of appendiceal mucocele. We report a relatively rare case of a giant appendiceal mucocele caused by mucinous cystadenocarcinoma, which occupied the entire abdomen of an adult woman.

**Case presentation:**

A 63-year-old woman presented with a chief complaint of abdominal distention. Imaging studies showed a giant cystic mass occupying her entire abdomen. Laparotomy confirmed a giant appendiceal mucocele, and the patient underwent ileocecal resection. A mucinous deposit was not found in her abdominal cavity, and the ovaries were grossly normal bilaterally. The pathological diagnosis was mucinous adenocarcinoma with a low-grade mucinous neoplasm that invaded the subserosa. Regional lymph node metastasis was not found. She has had recurrence-free survival for 5 years.

**Conclusions:**

The present case is the largest appendiceal cystadenocarcinoma ever reported. The optimal treatment of an appendiceal neoplasm requires further research based on consensus terminology of an appendiceal mucocele.

## Background

Mucinous cystadenocarcinoma is the second most common etiology of appendiceal mucocele [1, 2], and it is usually a well-differentiated, slowly progressive neoplasm. The gross morphology is indistinguishable from other types of mucoceles, but it is easy to perforate them before a giant cystic tumor is produced. We report a relatively rare case of a giant appendiceal mucocele caused by mucinous cystadenocarcinoma, which occupied the entire abdomen of an adult woman.

## Case presentation

A previously healthy 63-year-old woman presented with a chief complaint of abdominal distention. She denied abdominal pain, nausea, or constipation. She also did not have any relevant medical history. Her abdomen was remarkably distended but was soft and nontender. Blood test indicated slight anemia of hemoglobin (10.2 g/dL; normal range, 11.6–14.8 g/dL) and increased levels of the carcinoembryonic antigen (CEA) and cancer antigen 19-9 (CA19-9) to 406.1 ng/mL (normal range, 0–5.0 ng/mL) and 66 U/mL (normal range, 0–37.0 U/mL), respectively. The CA-125 level was normal.

**Fig. 1:**
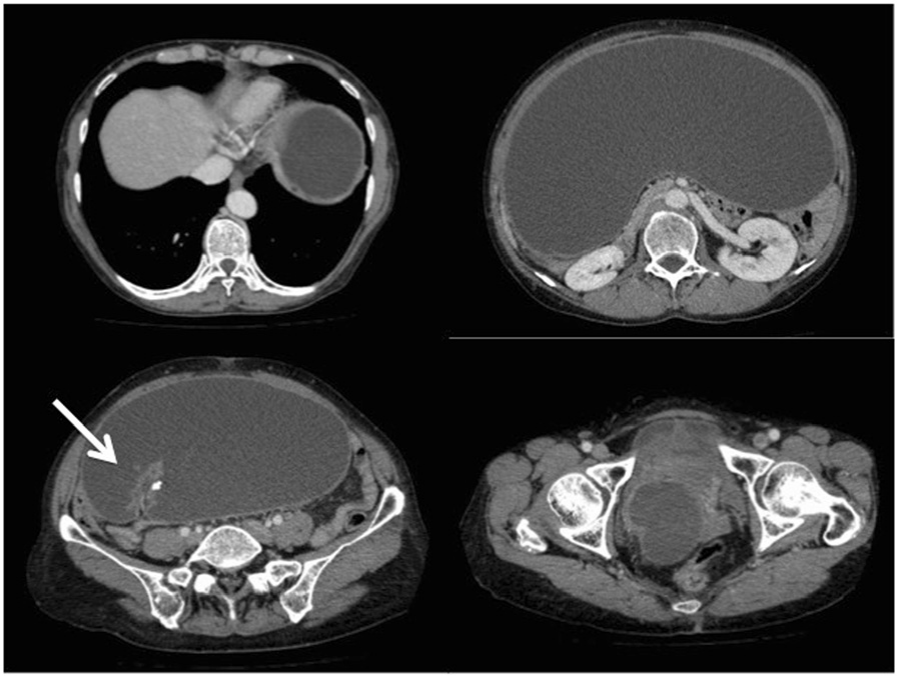
Contrast-enhanced computed tomography showed a well-encapsulated, unilocular cystic mass which occupied the entire abdomen from just below the diaphragm to the pelvis. *Arrow* showed a dendritic structure protruding into the tumor cavity

**Fig. 2:**
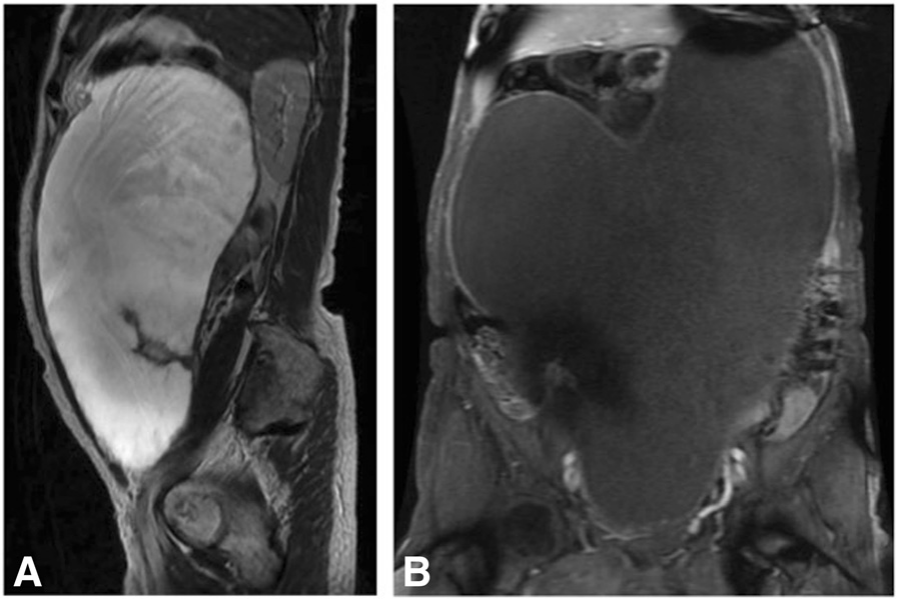
Magnetic resonance imaging demonstrated a giant unilocular cystic mass with high signal intensity on T2-weighted images (**a**) and intermediate signal intensity on T1-weighted images (**b**)

Contrast-enhanced computed tomography (CT) showed a well-encapsulated, unilocular cystic mass with focal mural calcification. It occupied her entire abdomen from just below the diaphragm to the pelvis, and it measured 40.2 cm in length (Fig. 1). Magnetic resonance imaging (MRI) demonstrated a cystic mass with high signal intensity on T2-weighted images and intermediate signal intensity on T1-weighted images (Fig. 2). These imaging studies also showed a dendritic structure protruding into the cavity, and the feeding artery originated from a branch of the ileocecal artery. Extrinsic obstruction due to the giant mass was identified on colonoscopy, but the orifice of the appendix remained intact. Based on these results, our preoperative diagnosis was a large neoplastic mucocele of the appendix.

Laparotomy showed the enlarged appendix, which occupied the entire abdomen. A mucinous deposit was not found in her abdominal cavity, and the ovaries were grossly normal bilaterally. Even though the cyst adhered strongly to the abdominal wall due to inflammation, ileocecal resection was performed uneventfully.

**Fig. 3:**
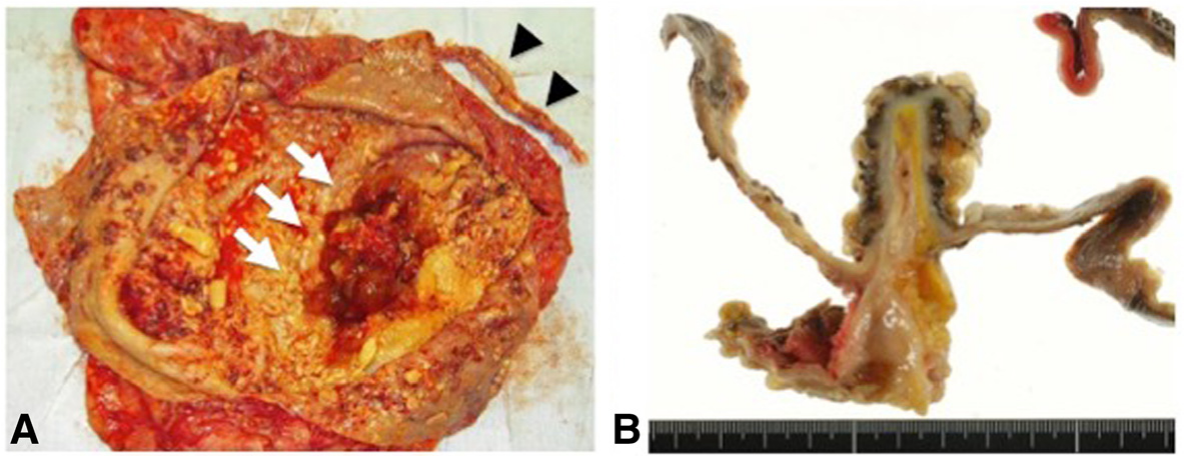
**a** A cross-section of the mass showed a cystically dilated lumen with the portion protruding from the appendiceal lumen (*arrow*). **b** A magnified view of the dendritic structure which appeared as a villous tumor

**Fig. 4:**
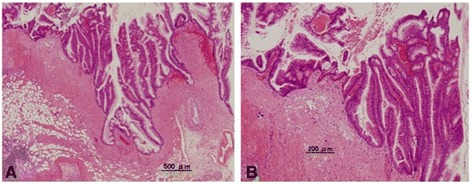
**a** Microscopical view of the tumor showed a mucinous adenocarcinoma, and a low-grade mucinous neoplasm is intermingled (hematoxylin and eosin stain, ×20). **b** Adenocarcinoma is observed to invade the subserosa of the appendix (hematoxylin and eosin stain, ×50)

The cystic mass contained about 7000 mL of serous fluid. The levels of CEA and CA19-9 in the fluid were 57,905 ng/mL and 1380 U/mL, respectively. Macroscopically, a cross-section of the mass showed a cystically dilated lumen containing mucin, and the portion protruding from the appendiceal lumen looked like a villous tumor (Fig. 3). The histopathological diagnosis was a mucinous adenocarcinoma with a low-grade mucinous neoplasm that had invaded the wall into the subserosa (Fig. 4). Regional lymph node metastasis was not detected. The levels of CEA and CA19-9 were within normal range 3 months postoperatively, and the patient has had recurrence-free survival for 5 years.

### Discussion

We report a rare case of a giant appendiceal mucocele caused by mucinous cystadenocarcinoma, which occupied the entire abdomen of an adult woman.

An appendiceal mucocele is a morphologic entity referring to the distention of the appendicular lumen due to the accumulation of mucus. It has been a long time since it was first described by Rokitansky [3], but the terminology for appendiceal mucocele has not been agreed on [4]. Mucoceles of the appendix have been classified into four types: (1) a simple mucocele or retention cyst resulting from obstruction of the appendiceal outflow, (2) mucosal hyperplasia, (3) a benign mucinous cystadenoma, and (4) a mucinous cystadenocarcinoma [5, 6]. A mucinous cystadenocarcinoma is the second most common etiology after mucinous cystadenoma, and it accounts for 11 to 20 % of cases of appendiceal mucoceles.

Although luminal dilatation is mild (up to 1 cm) in retention mucocele and hyperplasia, mucinous cystadenoma and cystadenocarcinoma often exhibit marked distention [7]. We searched PubMed for publications on mucoceles of the appendix with the keywords “giant” and “large,” and there have been about 50 cases reported from 1919 to 2015. The median diameter was 13 cm, and the largest mucocele ever reported was 40 cm in diameter, which was caused by a mucinous cystadenoma [8]. The majority of these giant mucoceles were caused by mucinous cystadenomas, whereas cystadenocarcinomas were the cause of only a few cases [9–14]. Therefore, the present case is rare in two respects: it is the largest mass among all types of appendiceal mucoceles, and it is a giant mucocele caused by a cystadenocarcinoma.

A mucocele is usually diagnosed by an abdominal CT scan. The typical finding of an appendiceal mucocele is a low attenuated, well-encapsulated, thin-walled cystic mass in the right lower quadrant. Mural calcification is seen in less than 50 % of cases [6]. MRI demonstrates a mass with intermediate signal intensity on T1-weighted images and homogeneous high signal intensity on T2-weighted images [15, 16]. An enhancing nodule in the mucocele wall is suggestive of a cystadenocarcinoma. In the present case, the dendritic structure within the cystic cavity was a clue to suspect malignancy.

However, the precise preoperative distinction between a cystadenoma and cystadenocarcinoma is often difficult [17, 18]. The tumor marker level is insufficient for an accurate diagnosis, because it is still unclear whether there is a difference in the serum or cystic fluid of CEA and CA19-9 levels between cystadenomas and cystadenocarcinomas [19, 20].

The boundary between cystadenomas and cystadenocarcinomas is ambiguous even in a pathological study. The term “mucinous tumor of low malignant potential” [21] or “low-grade appendiceal mucinous neoplasm” [22] was introduced to describe intermediate grades between cystadenomas and cystadenocarcinomas [23]. Furthermore, various subclassifications have been proposed according to the presence of acellular peritoneal mucin deposits or extra-appendiceal neoplastic epithelium [21, 22].

The Peritoneal Surface Oncology Group International recently reported the consensus for classification and pathologic reporting [24]. It argues that the term “cystadenoma” should no longer be used as a diagnostic term with regard to the appendix. Mucinous adenocarcinoma is defined as a mucinous tumor with infiltrative invasion. Mucinous neoplasm with low-grade cytologic atypia is classified as low-grade appendiceal mucinous neoplasm (LAMN) regardless of its associated features. The term “high-grade appendiceal mucinous neoplasm” was proposed for lesions with low-grade architectural features similar to LAMN but with high-grade cytologic features.

The operative strategy is also changing. It was the standard concept, and it still is in many facilities, that if the histological diagnosis is hyperplasia or cystadenoma, appendectomy is the definite treatment, but if it is a cystadenocarcinoma, resection should be combined with right colectomy [25, 26]. However, appendectomy combined with excision of all the mesoappendiceal fat can be adequate in patients with a cystadenocarcinoma in the absence of mesenteric adjacent organ or peritoneal involvement [27, 28]. Obviously, right hemicolectomy is advised if tumor clearance is required.

Formerly, an appendiceal mucocele was considered an indication for open surgery, but now, many surgeons think that laparoscopic appendectomy is a reasonable choice when treating a mucocele of the appendix [29]. However, extreme care is imperative to avoid underestimating the extent of the disease and prevent iatrogenic rupture and dispersion of mucus or epithelial cells into the peritoneal cavity during the surgical procedure. In the current case, we converted to open ileocecal resection to achieve curative operation without causing intraoperative rupture.

## Conclusions

We describe the case of an adult woman with the largest mucinous cystadenocarcinoma of the appendix that occupied the entire abdomen. The optimal treatment of appendiceal neoplasm requires further research based on consensus terminology of an appendiceal mucocele.

### Consent

Written informed consent was obtained from the patient for publication of this Case report and any accompanying images. A copy of the written consent is available for review by the Editor-in-Chief of this journal.

## Abbreviations

CA: cancer antigen
CEA: carcinoembryonic antigen
CT: computed tomography
LAMN: low-grade appendiceal mucinous neoplasm
MRI: magnetic resonance imaging
